# Development of a More Effective Mosquito Trapping Box for Vector Control

**DOI:** 10.1155/2018/6241703

**Published:** 2018-08-01

**Authors:** Tanawat Chaiphongpachara, Ploypailin Bunyuen, Kitthisak Khlaeo Chansukh

**Affiliations:** ^1^College of Allied Health Science, Suan Sunandha Rajabhat University, Thailand; ^2^Bachelor of Public Health, College of Allied Health Sciences, Suan Sunandha Rajabhat University, Thailand; ^3^Department of Applied Thai Traditional Medicine, College of Allied Health Sciences, Suan Sunandha Rajabhat University, Thailand

## Abstract

Mosquito-borne diseases are a major public health issue in nearly all tropical and subtropical countries, making vector control imperative. The mosquito trapping box is one type of mosquito traps that is popular in some areas because it is affordable, environmentally friendly, and easy to produce. This research investigated whether the effectiveness of the mosquito trapping box could be increased through the addition of various physical factors, including a wooden frame, black cotton cloth, a fan, carbon dioxide (CO_2_), and heat, by testing a range of box designs in the Samut Songkhram Province, Thailand, between December 2016 and January 2017. We found that trapping boxes constructed with* Pinus kesiya *wood caught more mosquitoes than those constructed with two other types of wood or aluminum. We also found that mosquito trapping boxes were more effective when more factors were added, although these differences were only significant for black cotton cloth and CO_2_. These findings will guide the future development of mosquito trapping boxes for effective mosquito control in other areas, helping to reduce the incidence of mosquito-borne diseases.

## 1. Introduction

Mosquito-borne diseases, such as Zika virus, malaria, dengue fever, Japanese encephalitis, chikungunya, and filariasis, are a major public health issue in nearly all tropical and subtropical countries, causing millions of deaths each year [1]. In Thailand, mosquito-borne diseases still remain important human health problems. Most mosquito-borne infectious diseases are caused by nocturnal mosquitoes, including* Culex *and* Anopheles* spp. [1]. For mosquito-borne disease outbreaks carried by nocturnal mosquitoes in Thailand, malaria is the most important and* Anopheles *spp. are a vector*. Anopheles dirus, An. minimus*, and* An. maculatus* are also recognized as significant malaria vectors [2], especially along international borders, while* An. epiroticus* is a critical vector in coastal areas [3].* Culex *mosquitoes is one of the genera carrying Japanese encephalitis and filariasis [1]. There are many species of* Culex *that cause these disease in Thailand, such as* Cx. quinquefasciatus*,* Cx. tritaeniorhynchus, Cx. gelidus*, and* Cx. sitiens *[4].

Currently, the most common method for controlling mosquitoes in people's homes is the use of chemical insecticides. However, the harmful effects of these chemicals on humans, other animals, and the environment [5], as well as the development of resistance to these chemicals by mosquitoes [6], are important issues. Therefore, the use of traps to reduce the number of adult mosquitoes is becoming increasingly popular [7].

Mosquito traps that are based on heat and odor are used in many communities. Female mosquitoes, which are the disease vectors, generally require a blood meal to provide sufficient nutrients for egg production [8] and thus are attracted to the heat and smell produced by humans or animals, being able to detect odorants such as carbon dioxide (CO_2_), lactic acid, and 1-octen-3-ol [9]. However, the performance of mosquito traps is affected by their layout and design.

The mosquito trapping box is one type of traps that is popular in some areas because it is easy to produce, affordable, and environmentally friendly. Recently, Pombi et al. [10] developed a sticky resting box for catching malaria vectors, which was generally found to be effective in collecting mosquitoes but had a varying performance between areas. Mosquito trapping boxes have also been developed in which other physical factors have been added, including CO_2_ [11], smell [12], and different component layouts [13], indicating that it may be possible to develop mosquito trapping boxes that are suited to a particular area.

The aim of this study was to develop an optimal trapping box for use in the Samut Songkhram Province, Thailand, where many mosquito species that are important disease vectors occur, including* Culex sitiens* and* Anopheles epiroticus *[14]. In particular, we examined the effects of the type of wood used, the inclusion of black cotton cloth, a fan, CO_2_, and heat on the number of mosquitoes caught. The results of this research will help in the development of mosquito odor-baited trapping boxes using local products as a cheap alternative for mosquito vector control.

## 2. Materials and Methods

### 2.1. Study Sites and Mosquito Collection

This study was carried out at a community dormitory in the district of Muang in Samut Songkhram Province, Thailand (13°24′32.52′′N 100°0′41.40′′E), where there is a high population density of people. Each trapping box was placed in a similar environment at a distance of 3 m from a house wall following Kweka et al. [15] (each stage of the development phase was conducted at the same sites) and was set for 12 hours from 18:00 to 06:00, which is the usual time that blood-sucking* Culex* and* Anopheles* mosquitoes are active. The trials were conducted between December 2016 and January 2017 and all trials were repeated with a Latin square sampling design (LSD). All the traps were rotated among every position at every sampling for each experiment (i.e., for each step of research and development).

### 2.2. Research and Development

We began this research and development study by reviewing the relevant literature and studying the vectors of mosquito-borne diseases in the study area. In this coastal area, there are two major mosquitoes, including* An. epiroticus* and* Cx. sitiens *[14]. Recently, there are reports of malaria parasite infections detected via* An. epiroticus* in Rayong province, Thailand.* An. epiroticus* has a biting pattern that increases from 6:00 to 8:00 PM and reaches a maximum at 12:00 PM (6.6 mosquitoes/person/hour) [3].* Cx. sitiens* has a biting cycle pattern that singly peaks at 7.00 to 8.00 PM and a maximum biting rate of 108 mosquitoes/person/hour [16]. In addition, both species of mosquitoes have bite behavior outdoors [3, 16]. Therefore, we adopted a plan to set box traps for mosquito collection at night and outside the home. We then entered the development phase of designing a suitable mosquito trapping box, which involved the sequential addition of various physical factors over five step, as outlined below.

S*tep 1: Selection of Wood for a Wooden Trapping Box*. In the first step of development, we constructed mosquito trapping boxes from three different types of strong-smelling wood (*Dipterocarpus alatus*,* Tectona grandis*, and* Pinus kesiya*) purchased from a wood shop in Phetchabun Province and compared their performance.

**Figure 1:**
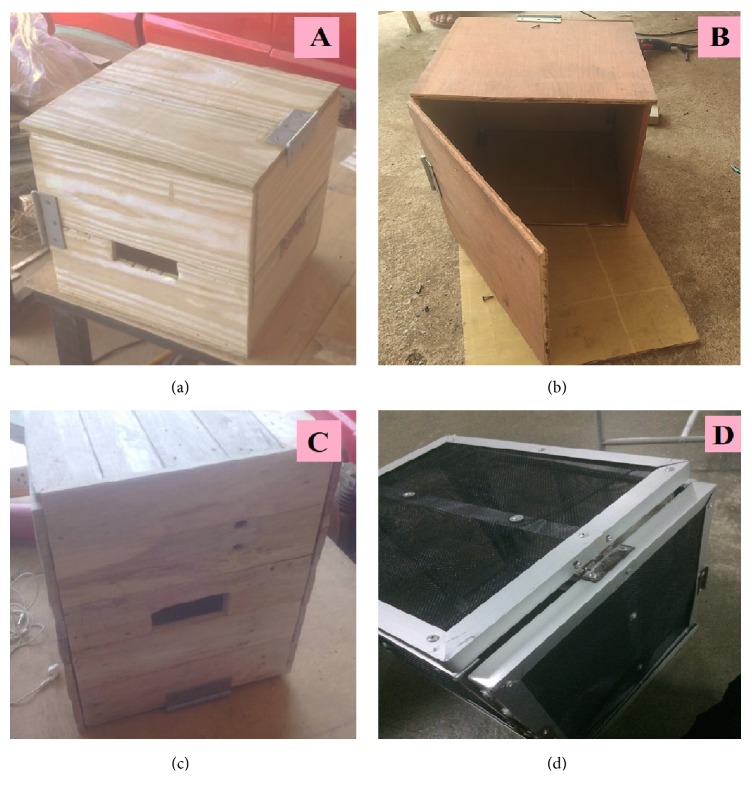
Design of the mosquito trapping boxes tested in Step 1 of the research and development phase. (a) Wooden trapping box constructed from* Pinus kesiya* wood; (b) wooden trapping box constructed from* Dipterocarpus alatus* wood; (c) wooden trapping box constructed from* Tectona grandis* wood; (d) control trapping box constructed from aluminum and black plastic mesh.

Each trapping box was a cube (30 × 30 × 30 cm) to make it easier to carry. Openings for mosquito entry measuring 10 cm long and 4 cm high were located in the middle of two of the side walls and upward-directed cloth barriers were attached to the walls at an angle of 45 degrees to prevent trapped mosquitoes from escaping, following Okumu et al. [13]. The top cover could be opened to remove mosquitoes and the trapping box could also be folded. In addition, a trapping box that was the same size as the experimental boxes but had an aluminum frame covered with a black mesh was used as a control. The design details of each trapping box are shown in Figure 1.

We set four mosquito trapping boxes (one per type of wood and an aluminum box as a control) for 12 hours (18:00–06:00) over consecutive nights, with no testing being carried out if it rained. We then transported the trapping boxes to a laboratory at the College of Allied Health Science, Suan Sunandha Rajabhat University, Samut Songkhram Education Center, Thailand, in the early morning and placed them at −80°C for 20 minutes to kill the mosquitoes. The trapped mosquitoes were identified using Illustrated keys to the mosquitoes of Thailand [17] and then counted.

S*tep 2: Addition of Black Cotton Cloth to the Wooden Trapping Box Designed in *S*tep 1*. In the second step of development, we took the wooden mosquito trapping box that was most effective in Step 1 and incorporated black cotton cloth into its design as it has previously been shown that black objects can attract mosquitoes [18].

**Figure 2:**
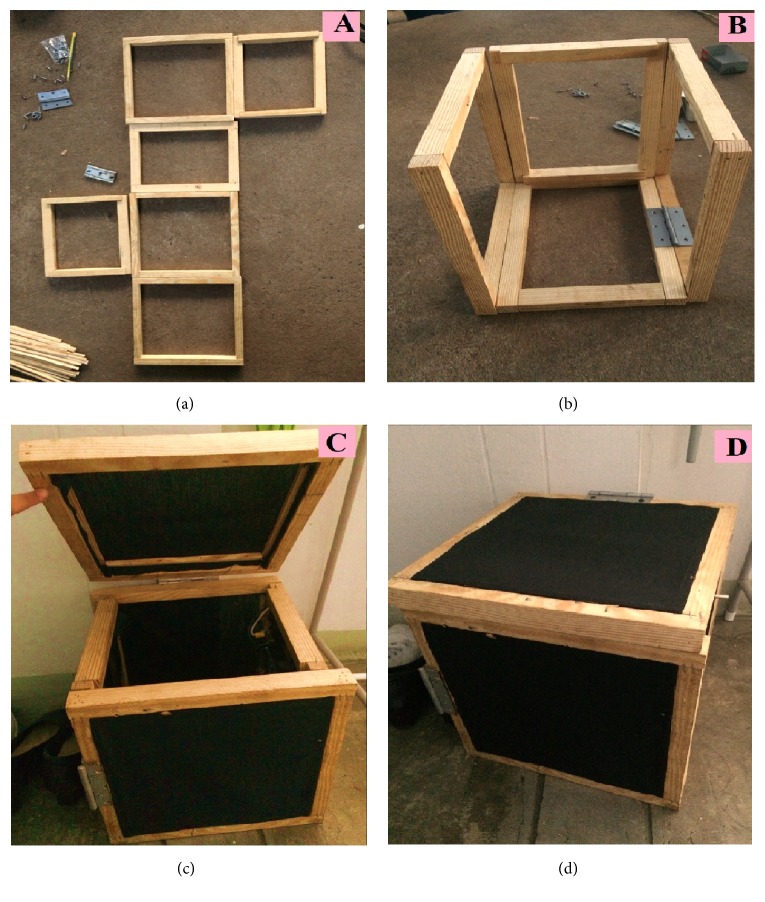
Design of the mosquito trapping box tested in Step 2 of the research and development phase. (a) A wooden frame was constructed using tacks; (b) the wooden frame was then used to make a square box with an opening lid for mosquito removal; (c) black cotton cloth was attached to the wooden frame; (d) appearance of the final wooden trapping box combined with black cotton cloth.

Black cloth (24 × 24 cm) was attached to each side of a wooden frame measuring 6 cm wide × 6 cm long × 30 cm high using tacks (Figure 2). Openings for the entrance of mosquitoes were again placed in the middle of two sides of the trapping box, as in Step 1. This box was then set alongside the most successful wooden trapping box from Step 1 and the aluminum trapping box with black plastic mesh as controls. The same trapping conditions and collection and identification methods as in Step 1 were used. 

*Step 3: Addition of a Fan to the Wooden Trapping Box Designed in Step 2*. In Step 3 of development, we took the trapping box that was constructed in Step 2 and incorporated a fan into its design.

**Figure 3:**
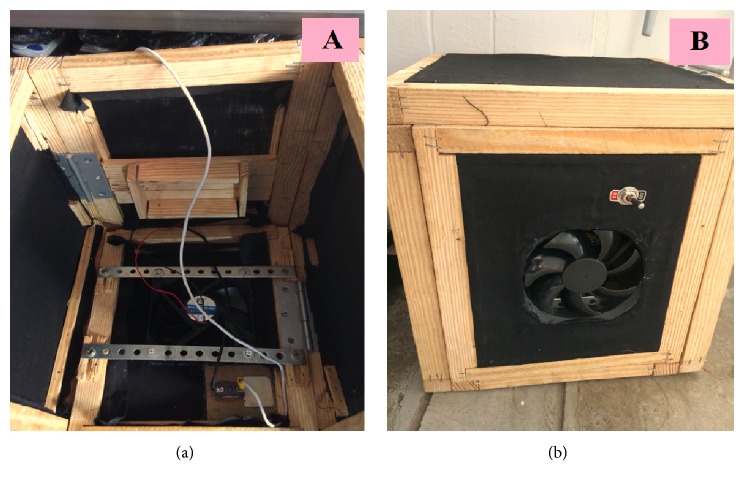
Design of the mosquito trapping box tested in Step 3 of the research and development phase. (a) Inside view; (b) outside view.

A black plastic fan (7 cm diameter, seven blades) that was powered by a rechargeable 6-V, 12-Ah battery was installed in one side of the trapping box that was developed in Step 2. A black bag was connected to the fan to collect any mosquitoes that were sucked in and a switching system for starting and stopping the fan was connected to the fan for convenience (Figure 3). This box was then set alongside the most successful wooden trapping box from Step 1, the modified trapping box designed in Step 2, and a trapping box made from aluminum and black plastic mesh as controls. The same trapping conditions and collection and identification methods were used as in Step 1, except that the mosquito bag was transported to the laboratory rather than the entire trap for the Step 3 trap.

*Step 4: Addition of CO*_*2*_ *to the Mosquito Trapping Box Designed in Step 3*. In Step 4 of development, we took the mosquito trapping box designed in Step 3 and added CO_2_ from dry ice.

Dry ice was added to an insulated dry ice container and placed on the side of the mosquito trapping box designed in Step 3. This box was then set alongside the most successful wooden trapping box from Step 1, the modified trapping boxes designed in Steps 2 and 3, and a trapping box made from aluminum and black plastic mesh as controls. The same trapping conditions and mosquito collection and identification methods were used as in Step 1.

*Step 5: Addition of Heat to the Mosquito Trapping Box Designed in Step 4*. In Step 5 of development, we took the mosquito trapping box that was designed in Step 4 and added heat from hand warmer.

A hand warmer (13 × 9.5 cm; Kobayashi, Japan), which had a temperature of approximately 55°C, was placed inside the mosquito trapping box designed in Step 4. This modified trap was then set alongside the most successful wooden trapping box from Step 1, the modified trapping boxes designed in Steps 2–4, and a trapping box made from aluminum and black plastic mesh as controls. The same trapping conditions and mosquito collection and identification methods as in Step 1 were used.

### 2.3. Statistical Analyses

We calculated the mean (± standard deviation [SD]) number of mosquitoes caught per trapping box in each step of development based on the number of mosquitoes caught per day. We then compared these values using Kruskal–Wallis Test followed by post hoc tests where a significant difference was detected at a 95% confidence level (p < 0.05).

## 3. Results

In this field experiment, we identified three mosquito species from two genera:* Culex quinquefasciatus* Say,* Culex sitiens* Weidemann, and* Anopheles epiroticus* Linton & Harbach. The findings from each of the trials are outlined below.

*Step 1: Selection of Wood*. Mosquito trapping boxes constructed from* P. kesiya *wood caught the largest number of mosquitoes (2.73 ± 0.61 mosquitoes per night per box) while those constructed from* D. alatus* wood and aluminum caught the least (0.53 ± 0.19 and 0.33 ± 0.08 mosquitoes, respectively) (Table 1). Statistical analysis further showed that* P. kesiya* boxes caught significantly more mosquitoes than the other two types of wooden boxes and the aluminum box (Table 2). Therefore, we used this type of wood for the frames of the trapping boxes designed in subsequent steps.

*Step 2: Addition of Black Cotton Cloth*. The mosquito trapping box constructed from* P. kesiya* wood combined with black cotton cloth attracted significantly more mosquitoes than the boxes constructed from* P. kesiya *wood without black cotton cloth and the aluminum box (12.25 ± 2.61 versus 2.25 ± 1.09 and 0.00 ± 0.00 mosquitoes, respectively) (Tables 1 and 2).

*Step 3: Addition of a Fan*. The mosquito trapping box constructed from the box designed in Step 2 combined with a black plastic fan attracted more mosquitoes than the* P. kesiya *wooden box used in Step 1, the wooden box with black cotton but no fan designed in Step 2, and the aluminum box (26.00 ± 5.00 versus 4.25 ± 1.15, 19.5 ± 5.16, and 0.00 ± 0.00 mosquitoes, respectively) (Table 1). However, these differences were only statistically significant for Step 1 box and the control (Table 2).

*Step 4: Addition of CO*_*2*_. The mosquito trapping box constructed from the box designed in Step 3 combined with CO_2_ attracted significantly more mosquitoes (41.00 ± 10.29 mosquitoes) than any of the other trapping boxes tested (Tables 1 and 2).

*Step 5: Addition of Heat*. The mosquito trapping box constructed from the box designed in Step 4 combined with a hand warmer as a source of heat attracted more mosquitoes (31.75 ± 7.41 mosquitoes) than any of the other trapping boxes tested (Table 1). However, these differences were only significant for Steps 1–3 boxes and the aluminum box (Table 2).

## 4. Discussion

We captured three species of mosquitoes that are important disease vectors in the study area:* An. epiroticus*, which can transmit malaria to humans [3], and* Cx. quinquefasciatus *and* Cx. sitiens*, which are vectors of filariasis and Japanese encephalitis [16, 19, 20]. Therefore, the control of these mosquitoes is very important to reduce the risk of mosquito-borne diseases in this area.

Mosquito trapping boxes are commonly used by communities to reduce the number of mosquitoes due to their ease of use. However, we require a better understanding of how various factors impact on the effectiveness of these traps. Since the densities of mosquitoes vary between different times of the day and across days, it is necessary to compare different trap designs at the same time. Therefore, in the present study, we made multiple comparisons during each step of development.

We first compared the effectiveness of trapping boxes constructed from three different types of wood, which showed that boxes made from* P. kesiya *wood caught significantly more mosquitoes than the other wooden and aluminum boxes. This may have been due to* P. kesiya* wood having a more distinct and stronger odor than the other woods tested. The olfactory system is very important for the survival of insects, including mosquitoes [21], as chemical cues, which are detected by olfactory sensory neurons, elicit odor-evoked behaviors, and changes in physiological state [22]. However, it should be noted that boxes constructed from* P. kesiya *wood still captured relatively few mosquitoes (2.73 ± 0.61 mosquitoes).

In Step 2 of development, we clearly showed that the use of black materials increases the efficiency of the mosquito trapping box. Color is perceived by photoreceptor molecules in the eyes [23] and there have been many previous reports showing that black can attract mosquitoes with high efficiency [24, 25]. Furthermore, it has been shown that the use of black cloth increases the ability of Ifakara odor-baited stations to lure mosquitoes [13].

The installation of a fan in a trapping box increases the likelihood that mosquitoes will enter the box. Consequently, a fan is an important component of the Centers for Disease Control miniature light trap, which is the standard trap used in medical entomology, particularly for the collection of mosquito vectors [26, 27]. However, the addition of a fan to the trapping box in Step 3 of development did not significantly improve its effectiveness compared with the trap designed in Step 2.

Female mosquitoes usually require proteins from the blood of humans or animals for the development of their eggs [1]. To find their prey, mosquitoes follow the smell of CO_2_, which they detect using their antennae and maxillary palps [25]. We found that the addition of CO_2_ to the trapping box in Step 4 of development significantly increased its efficiency, matching the results of previous trials that have shown that CO_2_ greatly enhances the ability of various traps to catch mosquitoes [28, 29]. However, it should be noted that CO_2_ is difficult to use in the field because it requires dry ice and servicing of the trapping boxes each night.

Female mosquitoes are also attracted to heat [26], detecting the specific temperature of humans or animals to locate their prey [1]. However, although slightly more mosquitoes were captured by the trapping box that included a heat source designed in Step 5 of development than by the unheated trapping box developed in Step 4, this difference was not significant. Similarly, Olamga et al. [30] found that olfactory cues are important for host-seeking in* An. gambiae*, while heat plays a minor role. For the number of mosquitoes that are trapped classified by species, we found the most* Cx. quinquefasciatus *during all steps of development, especially in Steps 4-5 with a number more than other species prominently. Consistent with previous research, it has been reported that CO2 can attract more* Cx. quinquefasciatus* mosquitoes [28].

Our mosquito trapping box in this study was designed based on resting traps that capture female mosquitoes; they must find a suitable place to rest after feeding on blood. However, we have increased the efficiency to make mosquitoes more responsive. Recently, there were reports that the smell of bait increases the efficiency of the resting traps in attracting mosquitoes. Kweka et al. [15] used wet black cotton cloth moistened with fresh cattle urine in a resting box and Logita et al. [29] utilized odor bait in a sticky trap to capture* Anopheles arabiensis*. The results of both studies have shown that odor bait can be effective in attracting mosquitoes. This is consistent with the results of our research that led to CO2 as an odor from the victim in nature and found it enhanced the efficiency of the mosquito trapping box. The reason for this may be from two behaviors of mosquitoes, including finding places for resting and determining a victim for biting.

## 5. Conclusions

In this study, we found that the efficiency of mosquito trapping boxes tends to increase as more physical factors are added to their design. However, the features that can be included will depend on the areas in which trapping is being conducted, as it may not be possible to include dry ice, a heat source, or a fan in some places. The findings of this research will guide the development of mosquito trapping boxes as a cheap, easy to manufacture, and environmentally friendly option for mosquito control in other areas, helping to reduce the incidence of mosquito-borne diseases. Although development in higher steps of mosquito trapping box may make it more expensive and closer to a traditional trap, like a CDC Miniature Light Trap or a BG Sentinel, these developments are much cheaper than standard traps, which are not available in Thailand and must be imported from abroad.

## Figures and Tables

**Table 1:** Mean number of mosquitoes caught by each type of trapping box in steps of development.

S	Factors tested	Number of mosquitoes caught per night (mean ± SD)						Total
		*An. epiroticus*		*Cx. quinquefasciatus*		*Cx. sitiens*		
		Male	Female	Male	Female	Male	Female	
1	Type of wood:							
	*P. kesiya* wood	0.00±0.00	0.33±0.63	0.07±0.26	1.80±1.93	0.00±0.00	0.53±0.74	2.73±0.61
	*D. alatus* wood	0.00±0.00	0.00±0.00	0.07±0.26	0.46±0.91	0.00±0.00	0.00±0.00	0.53±0.19
	*T. grandis* wood	0.07±0.26	0.33±0.61	0.00±0.00	0.33±0.48	0.13±0.35	0.20±0.41	1.07±0.13
	Aluminum	0.00±0.00	0.07±0.26	0.06±0.26	0.20±0.56	0.00±0.00	0.00±0.00	0.33±0.08

2	Addition of black cotton:							
	PW + BC^*∗*^	0.25±0.50	2.75±2.22	1.25±0.95	7.00±4.54	0.00±0.00	1.00±1.41	12.25±2.61
	PW	0.00±0.00	0.25±0.50	0.00±0.00	2.00±2.16	0.00±0.00	0.00±0.00	2.25±1.09
	Aluminum	0.00±0.00	0.00±0.00	0.00±0.00	0.00±0.00	0.00±0.00	0.00±0.00	0.00±0.00

3	Addition of fan:							
	PW + BC + F^*∗*^	1.50±1.91	4.25±2.98	5.00±5.09	14.0±9.79	0.00±0.00	1.25±1.50	26.00±5.00
	PW + BC	0.00±0.00	1.50±1.91	3.25±2.36	13.5±8.66	0.00±0.00	1.25±1.50	19.5±5.16
	PW	0.25±0.50	0.25±0.50	0.75±0.95	3.00±3.46	0.00±0.00	0.00±0.00	4.25±1.15
	Aluminum	0.00±0.00	0.00±0.00	0.00±0.00	0.00±0.00	0.00±0.00	0.00±0.00	0.00±0.00

4	Addition of CO_2_:							
	PW + BC + F + CO_2_^*∗*^	0.50±0.57	3.75±1.50	5.50±2.38	27.50±6.45	0.75±0.95	3.00±1.82	41.00±10.29
	PW + BC + F	0.20±0.45	3.20±1.45	1.40±1.14	21.00±3.32	0.00±0.00	2.20±0.84	27.40±2.88
	PW + BC	0.25±0.50	1.75±0.50	4.75±2.87	15.5±3.10	0.00±0.00	1.00±1.15	23.25±5.94
	PW	0.25±0.50	0.75.±0.95	1.00±1.41	3.25±1.50	0.00±0.00	0.25±0.50	5.50±1.20
	Aluminum	0.00±0.00	0.00±0.00	0.00±0.00	0.25±0.50	0.00±0.00	0.00±0.00	0.25±0.10

5	Addition of heat:							
	PW + BC + F + CO_2_ + H^*∗*^	0.50±0.57	1.75±1.25	6.25±4.92	19.75±13.81	0.25±0.50	3.25±2.22	31.75±7.41
	PW + BC + F + CO_2_	0.60±0.55	1.80±1.10	2.20±0.84	21.00±9.59	0.20±0.45	2.40±0.55	30.20±7.76
	PW + BC + F	0.40±0.55	1.20±0.45	2.20±0.84	14.80±1.64	0.40±0.55	1.80±0.84	21.40±3.36
	PW + BC	0.25±0.50	1.75±1.25	3.25±2.75	9.75±6.65	0.25±0.50	0.75±0.95	16.00±3.65
	PW	0.00±0.00	0.50.±0.57	0.00±0.00	1.75±2.06	0.00±0.00	1.00±0.81	3.25±0.71
	Aluminum	0.00±0.00	0.00±0.00	0.00±0.00	0.50±1.00	0.00±0.00	0.00±0.00	2.00±0.81

^*∗*^ This mosquito trapping box was based on the box used in the previous development step.

S, step of development; SD, standard deviation; PW, wooden trapping box constructed from *P. kesiya* wood; BC, black cotton; F, fan; CO_2_, carbon dioxide; H, heat.

**Table 2:** Comparison of the mean number of mosquitoes caught by each type of trapping box in each step of development.

Step of development	Factors compared			Mean difference	*p*
1	*P. kesiya* wood	vs.	*D. alatus* wood	2.20	<0.001^*∗*^
			*T. grandis* wood	1.66	0.001^*∗*^
			Aluminum	2.40	<0.001^*∗*^
	*D. alatus* wood	vs.	*P. kesiya* wood	−2.20	<0.001^*∗*^
			*T. grandis* wood	−0.54	0.044^*∗*^
			Aluminum	0.20	1.000
	*T. grandis* wood	vs.	*P. kesiya* wood	−1.66	0.001^*∗*^
			*D. alatus* wood	0.54	0.044^*∗*^
			Aluminum	0.74	0.044^*∗*^

2	PW + BC	vs.	PW	10.00	0.013^*∗*^
			Aluminum	12.25	0.004^*∗*^

3	PW + BC + F	vs.	PW + BC	6.50	0.186
			PW	21.75	0.004^*∗*^
			Aluminum	26.00	0.001^*∗*^

4	PW + BC + F + CO_2_	vs.	PW + BC + F	13.60	0.001^*∗*^
			PW + BC	17.75	<0.001^*∗*^
			PW	35.50	<0.001^*∗*^
			Aluminum	40.75	<0.001^*∗*^

5	PW + BC + F + CO_2_ + H	vs.	PW + BC + F + CO^2^	1.55	0.086
			PW + BC + F	10.35	<0.001^*∗*^
			PW + BC	15.75	<0.001^*∗*^
			PW	28.50	<0.001^*∗*^
			Aluminum	31.75	<0.001^*∗*^

^*∗*^Significant at the 0.05 level (one-way analysis of variance). PW, wooden trapping box constructed from *P. kesiya *wood; BC, black cotton; F, fan; CO_2_, carbon dioxide; H, heat.

## Data Availability

The data supporting the conclusions of this article are provided within the article. The datasets generated and analyzed during the current study are available from the corresponding author upon reasonable request.
